# Development and validation of a hypoxia-related gene signature to predict overall survival in early-stage lung adenocarcinoma patients

**DOI:** 10.1177/1758835920937904

**Published:** 2020-07-02

**Authors:** Jing Sun, Tianyu Zhao, Di Zhao, Xin Qi, Xuanwen Bao, Run Shi, Chuan Su

**Affiliations:** Department of Internal Medicine IV, University Hospital, Ludwig Maximilian University of Munich, Munich, Germany; Institute and Clinic for Occupational, Social and Environmental Medicine, University Hospital, Ludwig Maximilian University of Munich; Comprehensive Pneumology Center (CPC) Munich, Member DZL; German Center for Lung Research, Munich, Germany; Institute of Epidemiology, Helmholtz Zentrum München, German Research Center for Environmental Health, Neuherberg, Germany; Department of Cardiology, The First Affiliated Hospital of Nanjing Medical University, Nanjing, China; State Key Lab of Reproductive Medicine, Department of Pathogen Biology and Immunology, Jiangsu Province Key Laboratory of Pathogen Biology, Center for Global Health, Nanjing Medical University, Nanjing, Jiangsu, China; Institute of Radiation Biology, Helmholtz Center Munich, German Research Center for Environmental Health, Neuherberg, Germany; Technical University of Munich, Munich, Germany; Department of Radiation Oncology, University Hospital, Ludwig Maximilian University of Munich, Marchioninistr. No.15, Munich, Bayern 81377, Germany; State Key Lab of Reproductive Medicine, Department of Pathogen Biology and Immunology, Jiangsu Province Key Laboratory of Pathogen Biology, Center for Global Health, Nanjing Medical University, 101 Longmian Avenue, Jiangning District, Nanjing, Jiangsu 211166, China

**Keywords:** early-stage lung adenocarcinoma, gene signature, hypoxia, prognosis, therapeutic resistance

## Abstract

**Background:**

Patients with early-stage lung adenocarcinoma (LUAD) exhibit significant heterogeneity in overall survival. The current tumour-node-metastasis staging system is insufficient to provide precise prediction for prognosis.

**Methods:**

We quantified the levels of various hallmarks of cancer and identified hypoxia as the primary risk factor for overall survival in early-stage LUAD. Different bioinformatic and statistical methods were combined to construct a robust hypoxia-related gene signature for prognosis. Furthermore, a decision tree and a nomogram were constructed based on the gene signature and clinicopathological features to improve risk stratification and quantify risk assessment for individual patients.

**Results:**

The hypoxia-related gene signature discriminated high-risk patients at an early stage in our investigated cohorts. Survival analyses demonstrated that our gene signature served as an independent risk factor for overall survival. The decision tree identified risk subgroups powerfully, and the nomogram exhibited high accuracy.

**Conclusions:**

Our study might contribute to the optimization of risk stratification for survival and personalized management of early-stage LUAD.

## Introduction

Lung adenocarcinoma (LUAD) is the most common subtype of non-small cell lung cancer (NSCLC).^1^ Currently, treatment decisions for individual LUAD patients are based mainly on patient- and cancer-specific factors, such as tumour-node-metastasis (TNM) staging and differentiation grade. However, the predictive power and accuracy for prognosis are often insufficient. Thus, reliable predictors that can accurately estimate prognosis would bring tremendous value in guiding the management of LUAD.^2^ For example, better classification of early-stage LUAD after surgery should be used because several large randomized studies suggested that most patients who were sectioned as pathological stage I (*p*-stage I) and received adjuvant therapy showed no overall survival benefit among unselected patients.^3,4^ The 5-year overall survival remains unfavourable in patients with *p*-stage I, with a rate ranging from 73% in Ia to 58% in Ib.^5^ Therefore, in addition to traditional strategies, there is an urgent need to seek more accurate predictors for early-stage LUAD to discriminate high-risk subsets that could benefit from systemic treatment.

Hypoxia, or lack of oxygen, is a feature of most solid tumours.^6^ The hypoxic environment in tumours is a result of an imbalance between decreased oxygen supply and increased oxygen demand, which is widely considered to be associated with resistance to therapies, advanced aggressiveness and poor clinical outcomes.^7–9^ Although several studies have indicated that intratumoural hypoxia and increased hypoxia-inducible factor 1-alpha (HIF1A) expression are firmly associated with cancer progression and poor survival in lung adenocarcinoma,^10–12^ no hypoxia-based method is available that can be used to identify high-risk patients in early stages.

In this study, we not only identified hypoxia among the various hallmarks of cancer as a dominant risk factor for overall survival in relatively early-stage (*p*-stage I and II) LUAD but also combined different methods to screen for robust biomarkers and establish a hypoxia-related gene signature for prognosis. In addition, we validated the prognostic value of the gene signature in four independent cohorts. Finally, an integrated model based on the gene signature and clinicopathological features was developed to improve the predictive power and accuracy.

## Material and methods

### Dataset preparation and data processing

A total of 1461 stage I–II LUAD patients with clinical annotations and follow-up information were included in our study across different platforms. The microarray dataset GSE72094 was downloaded from GEO (http://www.ncbi.nlm.nih.gov/geo/) and was used as the training set. This dataset was produced by a Rosetta/Merck Human RSTA Custom Affymetrix 2.0 microarray and contained 321 stage I–II LUAD patients meeting the criteria. Datasets GSE31210, GSE30219, GSE37745, GSE50081 and GSE29013 from the same chip platform (Affymetrix HG-U133 Plus 2.0 Array) were integrated into a new cohort and were used as the first validation set, which contained a total of 548 I–II LUAD patients meeting the criteria. All raw CEL files from the five datasets were downloaded and normalized using a robust multichip average (RMA) algorithm.^13^ Moreover, the RNA-Seq by Expectation-Maximization (RSEM) normalized RNA-seq data of 389 stage I–II LUAD patients were accessed from The Cancer Genome Atlas (TCGA) and were used as the second validation set. In addition, 111 LUAD patients from GSE42127 (Illumina HumanWG-6 v3.0 expression beadchip) and 92 LUAD patients from GSE13213 (Agilent-014850 Whole Human Genome Microarray 4 × 44K G4112F) were used as another two independent validation cohorts. The stage distribution in each cohort is shown in Supplemental Table S1. All microarray and RNA-seq data included in our study were normalized and log2 transformed.

### Candidate selection and signature establishment

In brief, the performances of cancer hallmarks in the training set were quantified by a single-sample gene set enrichment analysis (ssGSEA) algorithm (R package ‘gsva’) based on transcriptome profiling data and hallmark gene sets from the Molecular Signatures Database (MSigDB).^14,15^ A univariate Cox proportional-hazards (Cox-PH) regression model was used to evaluate the significance of different cancer hallmarks in early-stage LUAD using the R package ‘survival’. The package ‘wgcna’ (*weighted* gene co-expression *network analysis*) was used to construct a scale-free co-expression network and identify the module that was most correlated with hypoxia based on transcriptome profiling data and ssGSEA scores.^16^ Gene significance (GS) quantified the association of individual genes with hypoxia ssGSEA score, and module membership (MM) represented the correlation between module eigengenes and gene expression profiles. With a threshold of the *p* value of GS <0.0001 and the *p* value of univariate Cox regression <0.01, 211 candidates from the ‘hypoxia module’ were identified. Subsequently, a least absolute shrinkage and selection operator (LASSO) Cox regression model was used to further screen for the most robust prognostic markers.^17^ A hypoxia-related risk score (HRS) was established by including normalized gene expression values weighted by their LASSO Cox coefficients as follows:

$$\begin{array}{l} HRS=\sum_{i}Coefficient\left(mRNA_{i}\right)\\ \;\;\;\;\;\;\;\;\;\times Expression\left(mRNA_{i}\right). \end{array}$$

### Bioinformatic and statistical analyses

GSEA^18^ was performed to validate the hypoxic status in the high-HRS group with the gene set of hypoxia from MSigDB. IBM SPSS Statistics 20 (IBM Corp., Armonk, NY, USA), GraphPad Prism 8.0 (GraphPad Software Inc, San Diego, CA, USA), Stata 12 (StataCorp LLC, TX, USA) and R software (version 3.5.2, http://www.r-project.org) were used to analyse data and plot graphs. The Z-score method was used to normalize ssGSEA scores and HRS when necessary. The Kaplan–Meier method was used to draw survival curves, and the log-rank test was used to evaluate differences. A Cox proportional-hazards regression model was used to evaluate the significance of each parameter to overall survival. Time-dependent receiver operating characteristic (tROC) analysis was performed to measure the predictive power with the R package ‘survivalROC’,^19^ and the areas under the curve at different time points [AUC(t)] of all the variables were compared. Meta-analysis (*I*^2^ > 30%, random-effects model) was performed to evaluate the prognostic value in the pooled cohort. Non-negative matrix factorization (NMF) consensus clustering was used to divide one cohort without a full-scale gene signature expression pattern into different clusters according to the best k value with the R package ‘nmf’.^20^ Recursive partitioning analysis (RPA) was performed to construct a decision tree for risk stratification with the R package ‘rpart’.^21^ A nomogram and a calibration curve were plotted using the R package ‘rms’.^22^ Codes for important methods and algorithms involved in this study have been integrated and uploaded as a supplemental file. A webtool GSCALite (http://bioinfo.life.hust.edu.cn/web/GSCALite/) was used to analyse the relationships between IC50 data of different molecules and the gene signature expression profile in lung adenocarcinoma cell lines.^23^ Student’s *t* test or one-way analysis of variance was used to analyse differences between groups in variables with a normal distribution.

## Results

### Schematic diagram of the study design

**Figure 1.**
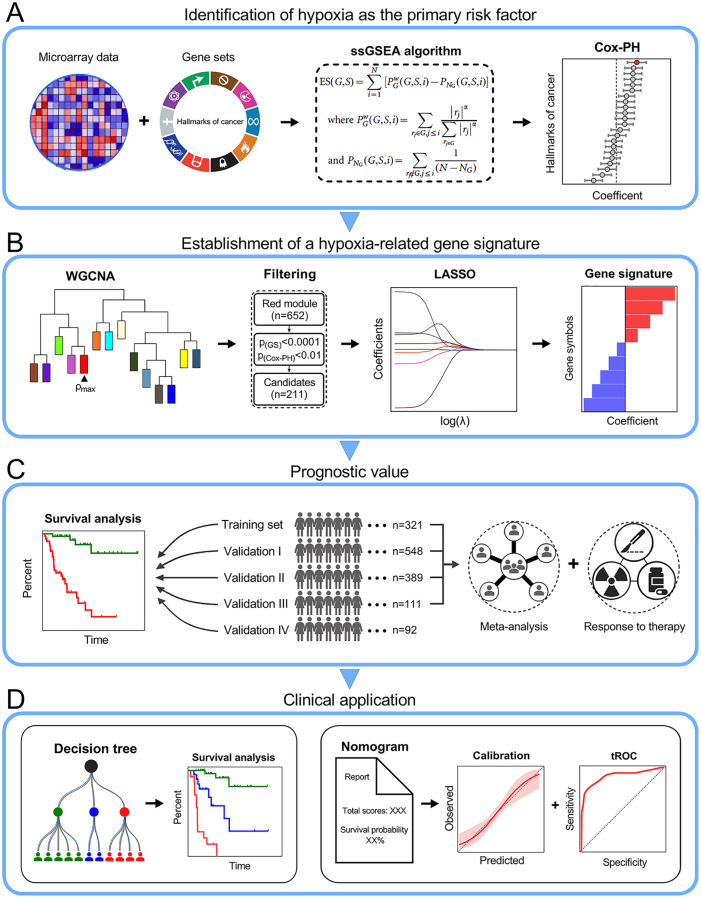
Schematic diagram of the study design. (A) Hypoxia was identified as the primary risk factor for overall survival in early-stage LUAD patients among various hallmarks of cancer. (B) Combined methods were used to establish a robust hypoxia-related gene signature for prognosis. (C) The prognostic value of the gene signature was validated in different cohorts. (D) Clinical application. Cox-PH, Cox proportional-hazards; LASSO, least absolute shrinkage and selection operator; LUAD, lung adenocarcinoma; ssGSEA, single-sample gene set enrichment analysis; tROC, time-dependent receiver operating characteristic; WGCNA, *weighted* gene co-expression *network analysis*.

First, hypoxia was identified as the primary risk factor for overall survival in early-stage LUAD patients among various cancer hallmarks (Figure 1A). Then, WGCNA, univariate Cox regression analysis and the LASSO algorithm were combined to screen for promising candidates and establish a robust hypoxia-related gene signature to predict survival (Figure 1B). Subsequently, the prognostic value of the gene signature was evaluated in the training and four independent validation cohorts. In addition, meta-analysis was performed to further validate its prognostic power, and response to therapies was evaluated to investigate whether the gene signature is a promising marker for treatment outcome (Figure 1C). Finally, a decision tree was constructed to improve risk stratification for survival, and a nomogram was built based on HRS and other clinicopathological variables to quantify risk assessment and survival probability for individual patients (Figure 1D).

### Hypoxia is identified as the primary risk factor for overall survival in early-stage LUAD

**Figure 2.**
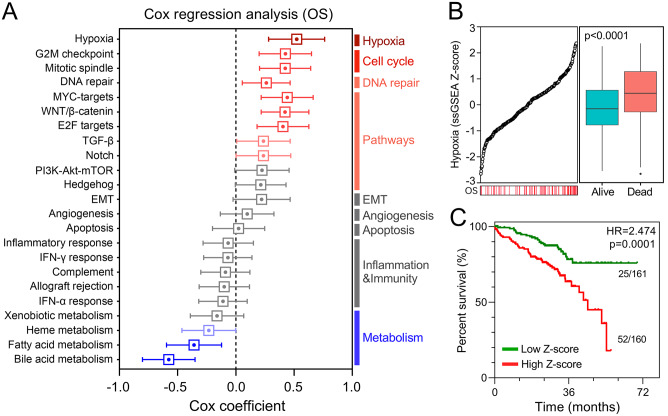
Hypoxia is identified as the primary risk factor for survival. (A) Univariate Cox regression analysis indicated that hypoxia was the primary risk factor among various hallmarks of cancer. (B) Hypoxia ssGSEA scores were significantly elevated in patients who died during follow up. (C) Kaplan–Meier analysis showed that patients with higher ssGSEA scores of hypoxia exhibited worse OS. EMT, epithelial–mesenchymal transition; IFN, interferon; OS, overall survival; PI3-Akt-mTOR, phosphatidylinositol-3-kinase-Akt-mammalian target of rapamycin; ssGSEA, single-sample gene set enrichment analysis; TGF, transforming growth factor.

Based on ssGSEA scores of cancer hallmarks and overall survival information in the training set, the Cox coefficient of each hallmark was calculated and ranked. Compared with other cancer hallmarks, such as the cell cycle, signalling pathways, epithelial–mesenchymal transition, angiogenesis, apoptosis, etc., hypoxia exhibited the most powerful effect on survival (Figure 2A). Figure 2B shows that Z-scores of the hypoxia ssGSEA were significantly elevated in dead patients compared with living patients during follow up. In the training set, 321 patients were divided into two equal parts according to the median, and the high-Z-score group exhibited worse overall survival compared with the lower group, with hazard ratio (HR) = 2.474 and *p* = 0.0001 (Figure 2C).

### Establishment of a hypoxia-related gene signature for prognosis

**Figure 3.**
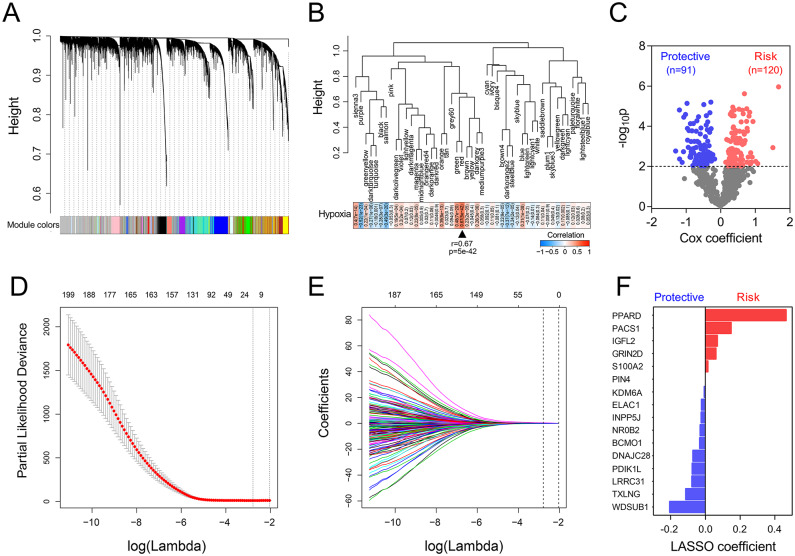
Establishment of a hypoxia-related gene signature. (A) WGCNA was performed with whole-transcriptome profiling data and hypoxia ssGSEA Z-scores. (B) A total of 47 non-grey modules were identified. The red module depicting the highest correlation (*r* = 0.67, *p* = 5e−42) was considered the most correlated with hypoxia. (C) A total of 211 promising candidates were identified among hub genes extracted from the red module. (D–E) The LASSO Cox regression model was used to identify the most robust markers, with an optimal λ value of 0.0617. (F) Distribution of LASSO coefficients of the hypoxia-related gene signature. LASSO, least absolute shrinkage and selection operator; ssGSEA, single-sample gene set enrichment analysis; WGCNA, *weighted* gene co-expression *network analysis*.

WGCNA was performed with whole-transcriptome profiling data and hypoxia ssGSEA Z-scores in the training set. With a power of *β* = 4 as the optimal soft threshold to ensure a scale-free co-expression network (Supplemental Figure S1), a total of 47 non-grey modules were generated (Figure 3A). Among these modules, the red module depicting the highest correlation (*r* = 0.67, *p* = 5e−42) was considered the most correlated with hypoxia (Figure 3B). With a threshold of *p* value for GS of <0.0001, hub genes extracted from the red module were submitted to univariate Cox regression analysis. With a threshold of *p* value for uni-Cox of <0.01, 211 promising candidates (91 protective and 120 risk markers) were identified (Figure 3C). Subsequently, the LASSO Cox regression model was used to identify the most robust markers for prognosis. Ten-fold cross-validation was applied to overcome over-fitting, with the optimal λ value of 0.0617 selected (Figure 3D). An ensemble of 16 genes (PPARD, PACS1, IGFL2, GRIN2D, S100A2, PIN4, KDM6A, ELAC1, INPP5J, NR0B2, BCMO1, DNAJC28, PDIK1L, LRRC31, TXLNG, WDSUB1) remained with their individual nonzero LASSO coefficients (Figure 3E), and the distribution of LASSO coefficients of the gene signature is shown in Figure 3F and Supplemental Table S2. Finally, the HRS formula was established as follows: ∑*i* *Coefficient* (*mRNA*_i_) × *Expression* (*mRNA*_i_). The expression level of each gene was log2 normalized.

### HRS serves as a risk factor for overall survival in each cohort

**Figure 4.**
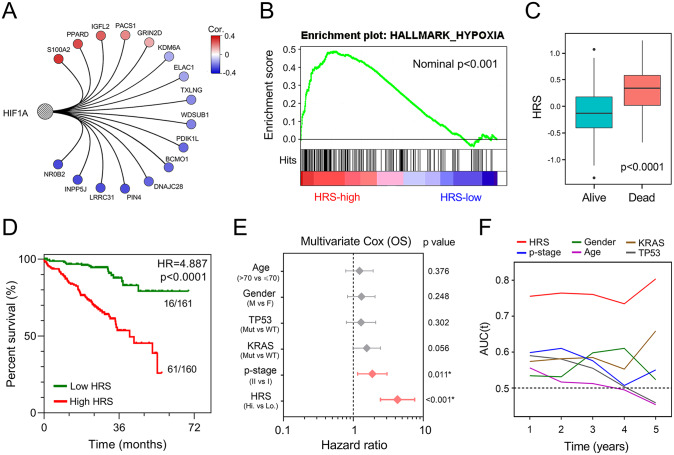
The gene signature predicts worse survival in the training set. (A) Correlations of the gene signature with HIF1A expression. (B) GSEA confirmed the hypoxic status in the high-HRS group. (C) HRS was significantly elevated in patients who died during follow-up. (D) Kaplan–Meier analysis showed that patients with higher HRS exhibited worse OS. (E) Multivariate Cox regression analysis demonstrated that HRS was an independent risk factor for OS. (F) tROC analysis showed that HRS was an accurate variable for survival prediction. GSEA, gene set enrichment analysis; HR, hazard ratio; HRS, hypoxia-related risk score; OS, overall survival; tROC, time-dependent receiver operating characteristic.

In the training set, five risk markers were shown to positively correlate with HIF1A expression, while the other 11 protective markers exhibited negative correlations with HIF1A (Figure 4A). With the gene set of hypoxia from MSigDB, GSEA confirmed the hypoxic status in the high-HRS group compared with the low-HRS group (Figure 4B). Compared with living patients, the risk score was significantly elevated in patients who died during follow up (Figure 4C). Kaplan–Meier analysis revealed that patients with higher HRS exhibited worse prognosis than those with lower scores (HR = 4.887, *p* < 0.0001, Figure 4D). Among various clinicopathological variables, multivariate Cox regression modelling demonstrated that AJCC TNM stage (HR = 1.8732, *p* = 0.011) and HRS (HR = 4.302, *p* < 0.001) were two independent risk factors for overall survival in the training cohort (Figure 4E). Furthermore, tROC analysis showed that HRS was the most accurate predictor for overall survival (Figure 4F).

**Figure 5.**
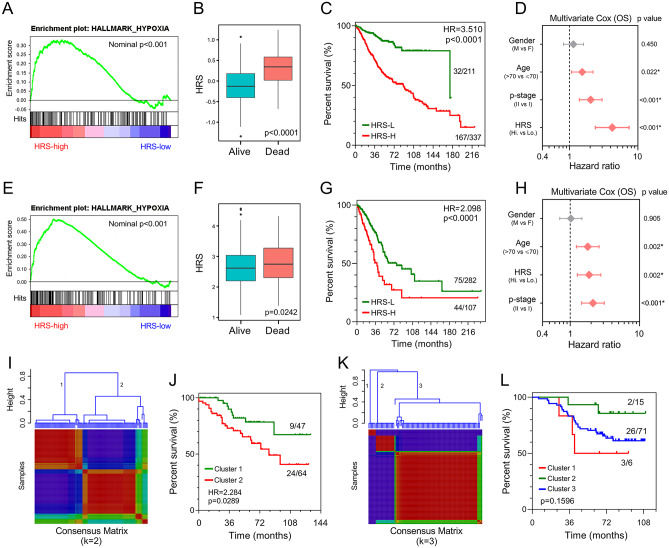
Validation of the gene signature in different series. (A, E) GSEA confirmed the hypoxic status in the validation I and II cohorts. (B, F) HRS was significantly elevated in deceased patients in the validation I and II cohorts. (C, G) Patients with higher HRS exhibited worse prognosis in the validation I and II cohorts. (D, H) Multivariate Cox regression analysis demonstrated that HRS was an independent risk factor for overall survival in the validation I and II cohorts. (I, K) The best k value was chosen for NMF consensus clustering in the validation III and IV cohorts. (J, L) Survival differed greatly in NMF-derived clusters based on the expression pattern of the gene signature. GSEA, gene set enrichment analysis; HR, hazard ratio; HRS, hypoxia-related risk score; NMF, non-negative matrix factorization); OS, overall survival.

To confirm the prognostic robustness of the hypoxia-related gene signature in different series, it was further validated in four independent external cohorts, which were described in the previous. Similarly, in the validation I and II cohorts, hypoxic status was confirmed in the high-HRS group with the hypoxia gene set using GSEA (Figure 5A, E), and HRS was significantly elevated in dead patients compared with living ones (I: *p* < 0.0001, Figure 5B; II: *p* = 0.0242, Figure 5F). Kaplan–Meier analysis demonstrated that high HRS predicted worse overall survival than lower HRS (I: HR = 3.510, *p* < 0.0001, Figure 5C; HR = 2.098, *p* < 0.0001, Figure 5G). Moreover, HRS acted as an independent risk factor for overall survival in multivariate Cox regression analysis (I: HR = 4.219, *p* < 0.001, Figure 5D; II: 1.874, *p* = 0.002, Figure 5H). In the validation III and IV cohorts, some genes included in the gene signature were missing due to the differences in platforms. Thus, NMF consensus clustering was used to divide one cohort into different groups according to the best k value based on the remaining expression pattern of the gene signature (Figure 5I, K). As shown in Figure 5J and L, overall survival differed between NMF-derived groups. Moreover, stage frequency in different signature groups or clusters in each cohort was shown in Supplemental Figure S2.

### HRS acts as an indicator of worse prognosis in the pooled cohort and a promising marker of therapeutic resistance

**Figure 6.**
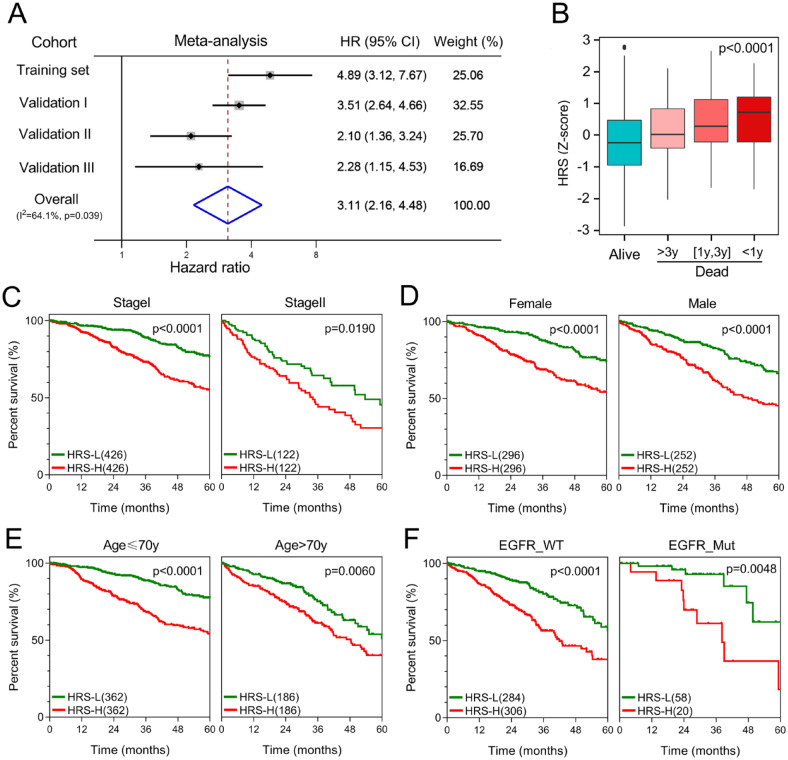
The gene signature serves as a valuable marker for poor survival in the pooled cohort and subgroups. (A) Meta-analysis. (B) HRS Z-scores were significantly elevated in deceased patients, especially in shorter-survival groups. (C–F) HRS discriminated high-risk patients in different clinicopathological including *p*-stage, gender, age and EGFR status subgroups. EGFR, epidermal growth factor receptor; HR, hazard ratio; HRS, hypoxia-related risk score; NMF, non-negative matrix factorization; OS, overall survival.

Meta-analysis was performed to analyse the prognostic value of the hypoxia-related gene signature in the pooled cohort integrating the training cohort and three validation cohorts, which were divided into two groups. As shown in Figure 6A, meta-analysis demonstrated that among the 1369 patients, those with higher HRS exhibited worse prognosis than those with lower HRS (pooled HR = 3.11, 95% CI 2.16–4.48). Then, 1096 patients with detailed TNM stage (Ia, Ib, IIa or IIb) and HRS were extracted for further investigation. HRS Z-scores were significantly elevated in those patients who died during follow up, especially in the shorter-survival groups (Figure 6B). HRS also discriminated high-risk patients with poor prognosis in different subgroups, including different clinicopathological features (sex, age and *p*-stage) and epidermal growth factor receptor (EGFR) mutation status (Figure 6C–F).

**Figure 7.**
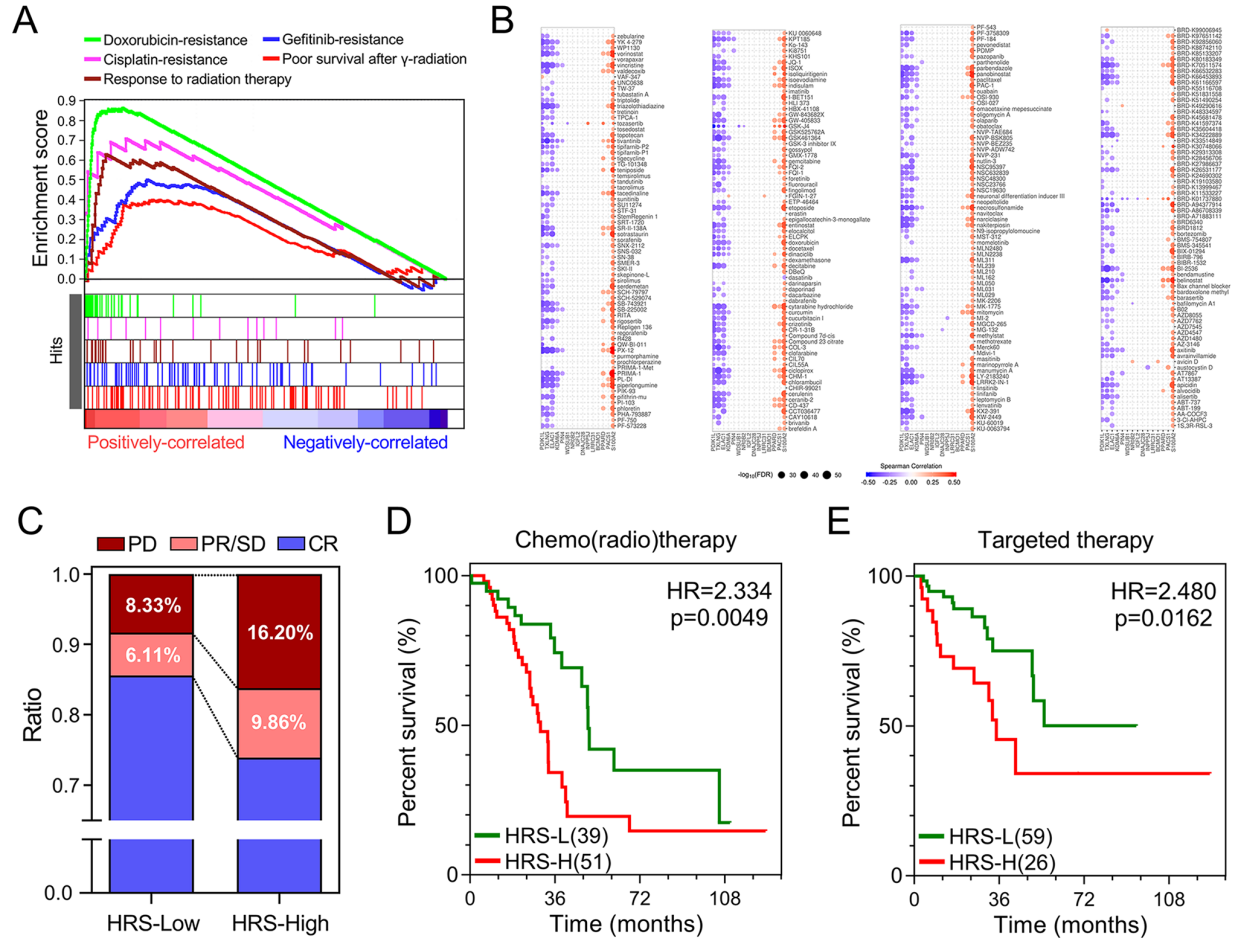
The gene signature serves as a promising marker of resistance to different treatments. (A) GSEA predicted that the gene signature was associated with therapeutic resistance. (B) A landscape plot was generated to depict the relationships between IC50 data of different molecules and the gene signature expression profile in LUAD cell lines. (C) The ratio of worse outcomes after surgery is greatly elevated in higher HRS group. (D, E) Patients with higher HRS exhibited worse OS among those who received adjuvant therapies including chemo(radio)therapy and targeted therapy. CR, complete remission; GSEA, gene set enrichment analysis; HR, hazard ratio; HRS, hypoxia-related risk score; IC50, half maximal inhibitory concentration; LUAD, lung adenocarcinoma; NMF, non-negative matrix factorization; OS, overall survival; PD, progressive disease; PR, partial remission, SD stable disease.

Considering tumour hypoxia always promotes resistance to therapy, we investigated whether the gene signature is a marker of therapeutic resistance. As shown in Figure 7A, GSEA predicted that higher HRS is significantly associated with resistance to different therapies, including chemotherapy, radiotherapy and targeted therapy in the training set. A landscape plot was generated by GSCALite to depict the relationships between drug responses and gene signature expression (Figure 7B). The bubble heatmap showed that some genes exhibited significant correlations with IC50 data in LUAD cell lines. In detail, S100A2, PACS1 and PPARD conferred drug resistance, while PDIK1L, TXLNG and ELAC1 exhibited drug sensitivity, which were consistent with the results in Figure 3F. Subsequently, treatment information and clinical outcomes from TCGA were used to validate the prediction. After initial treatment of surgery, the ratio of the status of progressive disease and partial remission or stable disease in higher HRS group was greatly elevated compared with lower HRS group (Figure 7C). Moreover, patients with higher HRS exhibited worse overall survival among those who received adjuvant therapies, including chemo(radio)therapy (HR = 2.334, *p* = 0.0049; Figure 7D) and targeted therapy (HR = 2.480, *p* = 0.0162; Figure 7E).

### Combination of the hypoxia signature and clinicopathological features improves risk stratification and survival prediction

**Figure 8.**
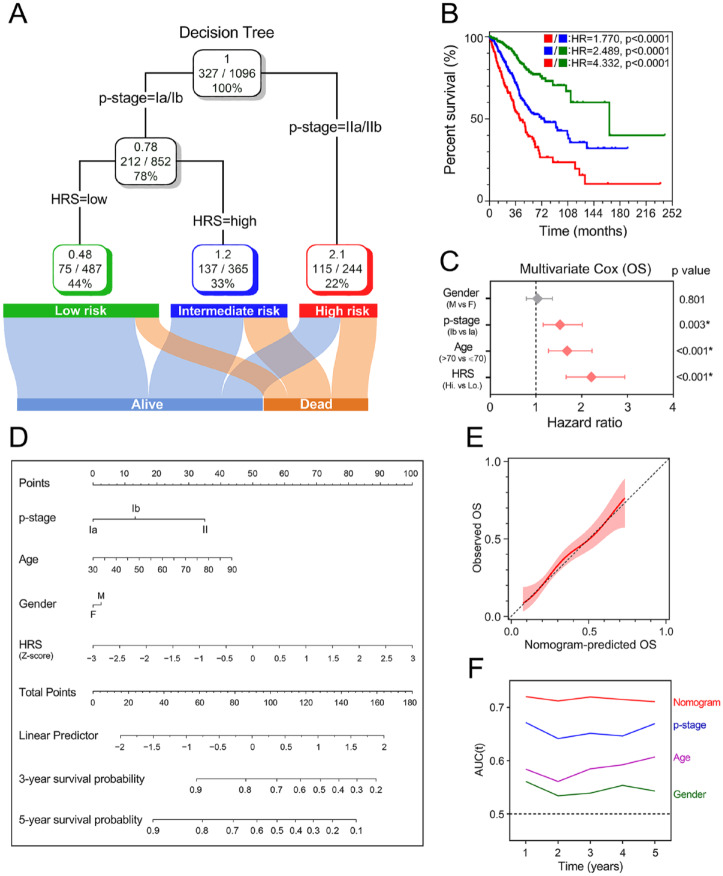
Combination of the hypoxia signature and clinicopathological features improves risk stratification and survival prediction. (A) A decision tree was constructed to improve risk stratification. (B) Performance of the decision tree. (C) Among *p*-stage I patients, HRS was the most important risk factor for OS. (D) A nomogram was constructed to quantify risk assessment for individual patients. (E) Calibration analysis indicated a high accuracy of survival prediction. (F) tROC analysis demonstrated that the nomogram was the most stable and powerful predictor for OS among all the clinical variables. HR, hazard ratio; HRS, hypoxia-related risk score; OS, overall survival; tROC, time-dependent receiver operating characteristic.

A total of 1096 patients with four parameters available, age (>70 or ⩽70), sex (male or female), TNM stage (Ia, Ib, IIa or IIb) and HRS (low or high), were used to construct a decision tree to improve risk stratification for overall survival. As shown in Figure 8A, only TNM stage and HRS remained in the decision tree, with three different risk subgroups identified. Interestingly, we observed that HRS replaced TNM staging in the node of stage I. As shown in the Kaplan–Meier curve in Figure 8B, overall survival differed markedly between the three risk subgroups. Among stage I patients, HRS was the most important predictor of overall survival in the multivariate Cox regression model (Figure 8C).

With the goal of quantifying the risk assessment and survival probability for individual LUAD patients in the early stage, a nomogram was built with HRS together with other clinicopathological features (Figure 8D). In the calibration analysis, the prediction line (red line and pink area) of the nomogram for 3-year survival probability was extremely close to the ideal performance (45-degree dotted line) (Figure 8E), suggesting a high level of accuracy of the nomogram. When compared with other features, the nomogram exhibited the most powerful and stable ability for survival prediction, with an average AUC above 0.7, much better than the pathological TNM staging (Ia, Ib, IIa and IIb) (Figure 8F).

## Discussion

Hypoxia, a hallmark of solid tumours, is a result of an imbalance between insufficient oxygen supply and increased oxygen demand associated with high proliferative rates.^24^ Tumour hypoxia has wide-ranging effects, causing various biological processes, such as metabolic alteration, angiogenesis, and metastasis.^25–27^ Significant crosstalk between hypoxia and other cancer hallmarks in solid cancer contributes to malignant progression and attenuated antitumour responses, leading to resistance to therapies and poor clinical outcomes.^28,29^ These observations indicate why hypoxia has prognostic value and why hypoxia has become an attractive therapeutic target.^30–32^ To date, some hypoxia gene signatures for prognosis have been developed in different cancer types, such as head and neck,^33^ breast,^34^ prostate,^35^ and bladder cancer.^36^ However, unavoidable deficiencies have marred previous studies. First, some of these hypoxia-related gene signatures were roughly established based on some literature-reported individual ‘hypoxia genes’, ignoring the fact that hypoxia is a cancer hallmark involving gene networks. Second, few established hypoxia-related molecular signatures have been integrated with the traditional prognostic system to optimize the clinical routine.

In this study, among various hallmarks of cancer, we identified hypoxia as the primary risk factor for overall survival using ssGSEA and Cox-PH regression models in relative early-stage (*p*-stage I and II) LUAD, which lacks reliable predictors for prognosis. WGCNA was performed to identify hypoxia-related gene modules based on transcriptome profiling data, and a univariate and LASSO Cox regression model was used to screen robust prognostic biomarkers to establish a hypoxia-related gene signature. The risk score derived from the hypoxia-related gene signature is called the HRS in our study. Subsequently, the prognostic value of the gene signature was validated in four independent cohorts across different platforms. In the meta-analysis and subgroup analysis, HRS still had the capacity to discriminate high-risk patients, suggesting it can serve as a reliable risk factor in pooled populations and similar-stage subgroups. In addition, in the adjuvant therapy groups, patients with higher HRS exhibited worse survival compared with lower-HRS patients, which might have resulted from the gene signature-derived resistance to therapies, indicating the gene signature also serves as a promising marker of therapeutic resistance in early-stage LUAD patients.

A decision tree was constructed to improve risk stratification in combination with clinicopathological features. In the decision tree, TNM staging still served as the major determinant. However, in the *p*-stage I node, the decision tree indicated that the risk stratification would be improved if the Ia/Ib staging was replaced with HRS. Moreover, in the pooled *p*-stage I cohort, the multivariate Cox regression analysis showed that HRS exhibited a considerable power of risk prediction for overall survival, even more significant than age and *p*-stage (Ia or Ib). The decision tree and multivariate Cox results jointly suggest that the hypoxia gene signature truly serves as a powerful risk factor for overall survival in early-stage LUAD patients, especially in *p*-stage I. To quantify the risk assessment for individual patients, a nomogram was generated including HRS with other clinicopathological features. Calibration analysis revealed that the nomogram exhibited an accurate prediction that was extremely close to actual survival. In addition, compared with any other single variable, tROC analysis demonstrated that the nomogram had the most stable and powerful ability for survival prediction at different time points during follow up.

Some biomarkers involved in our gene signature have been studied in many cancers, but most of them are rarely investigated in tumour hypoxia. For example, PPARD, a biomarker with the largest risk coefficient in our study, has been widely studied in various cancers, with comprehensive oncogenic functions to promote tumourigenesis, proliferation and metastasis.^37–39^ S100A2 induces metastasis in NSCLC and was reported as a prognostic marker for patients with stage I NSCLC.^40,41^ KDM6A, a histone demethylase, served as a protective biomarker in our study. KDM6A directly senses oxygen to control chromatin and cell fate,^42^ and loss of KDM6A contributes to the malignant phenotype by amplifying PRC2-regulated transcriptional repression in bladder cancer and conferring drug resistance in acute myeloid leukaemia.^43,44^ In summary, the biological functions associated with tumour hypoxia of the novel gene signature still require further investigation in LUAD.

Some limitations to our study should be acknowledged. First, this is a retrospective study, so the prognostic robustness and clinical usefulness of the hypoxia-related gene signature need further validation in larger prospective trials. Second, further experimental studies are needed to elucidate tumour hypoxia-related biological functions underlying the gene signature in LUAD.

## Conclusion

In summary, we established a novel hypoxia-related gene signature to discriminate high-risk patients with early-stage LUAD. Integrating this with clinicopathological features, we constructed a decision tree to optimize risk stratification for overall survival and a nomogram to quantify risk assessment for individual patients. The hypoxia gene signature-based model could be a useful tool to select high-risk early-stage patients who may benefit from adjuvant therapies and thus to facilitate personalized management of LUAD.

## Supplemental Material

Supplementary_figure_1 – Supplemental material for Development and validation of a hypoxia-related gene signature to predict overall survival in early-stage lung adenocarcinoma patients Click here for additional data file. Supplemental material, Supplementary_figure_1 for Development and validation of a hypoxia-related gene signature to predict overall survival in early-stage lung adenocarcinoma patients by Jing Sun, Tianyu Zhao, Di Zhao, Xin Qi, Xuanwen Bao, Run Shi and Chuan Su in Therapeutic Advances in Medical Oncology

Supplementary_figure_2 – Supplemental material for Development and validation of a hypoxia-related gene signature to predict overall survival in early-stage lung adenocarcinoma patients Click here for additional data file. Supplemental material, Supplementary_figure_2 for Development and validation of a hypoxia-related gene signature to predict overall survival in early-stage lung adenocarcinoma patients by Jing Sun, Tianyu Zhao, Di Zhao, Xin Qi, Xuanwen Bao, Run Shi and Chuan Su in Therapeutic Advances in Medical Oncology

Supplementary_file_SourceCode_R – Supplemental material for Development and validation of a hypoxia-related gene signature to predict overall survival in early-stage lung adenocarcinoma patients Click here for additional data file. Supplemental material, Supplementary_file_SourceCode_R for Development and validation of a hypoxia-related gene signature to predict overall survival in early-stage lung adenocarcinoma patients by Jing Sun, Tianyu Zhao, Di Zhao, Xin Qi, Xuanwen Bao, Run Shi and Chuan Su in Therapeutic Advances in Medical Oncology

Supplementary_table_1 – Supplemental material for Development and validation of a hypoxia-related gene signature to predict overall survival in early-stage lung adenocarcinoma patients Click here for additional data file. Supplemental material, Supplementary_table_1 for Development and validation of a hypoxia-related gene signature to predict overall survival in early-stage lung adenocarcinoma patients by Jing Sun, Tianyu Zhao, Di Zhao, Xin Qi, Xuanwen Bao, Run Shi and Chuan Su in Therapeutic Advances in Medical Oncology

Supplementary_table_2 – Supplemental material for Development and validation of a hypoxia-related gene signature to predict overall survival in early-stage lung adenocarcinoma patients Click here for additional data file. Supplemental material, Supplementary_table_2 for Development and validation of a hypoxia-related gene signature to predict overall survival in early-stage lung adenocarcinoma patients by Jing Sun, Tianyu Zhao, Di Zhao, Xin Qi, Xuanwen Bao, Run Shi and Chuan Su in Therapeutic Advances in Medical Oncology
