# Supplementary figures and images for: Development and validation of a hypoxia-related gene signature to predict overall survival in early-stage lung adenocarcinoma patients
Source: Ther Adv Med Oncol. 2020 Jul 2;12:1758835920937904. doi: 10.1177/1758835920937904 (PMC7333486; doi:10.1177/1758835920937904)

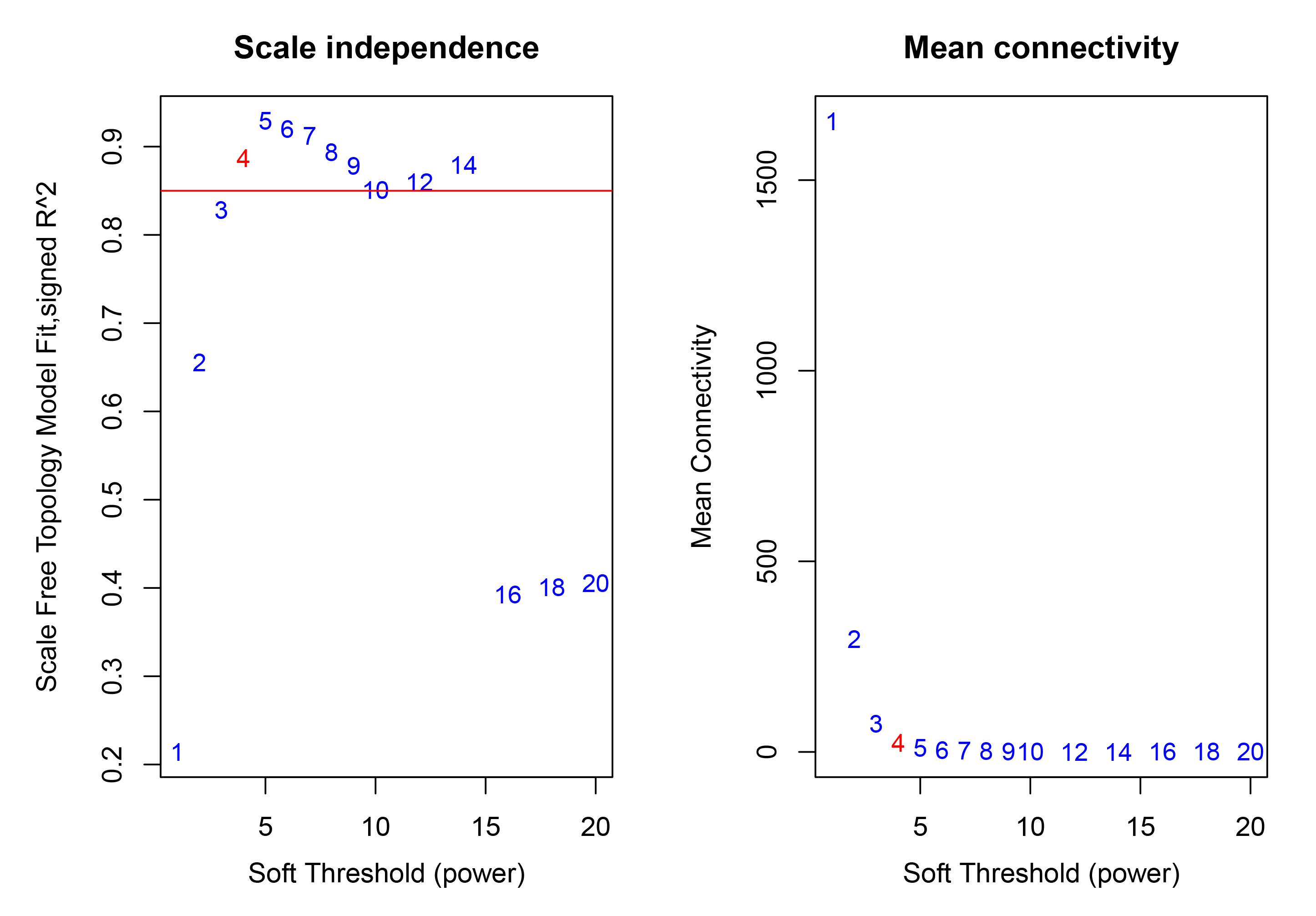

Supplement: Supplementary_figure_1 – Supplemental material for Development and validation of a hypoxia-related gene signature to predict overall survival in early-stage lung adenocarcinoma patients [file Supplementary_figure_1.tif]

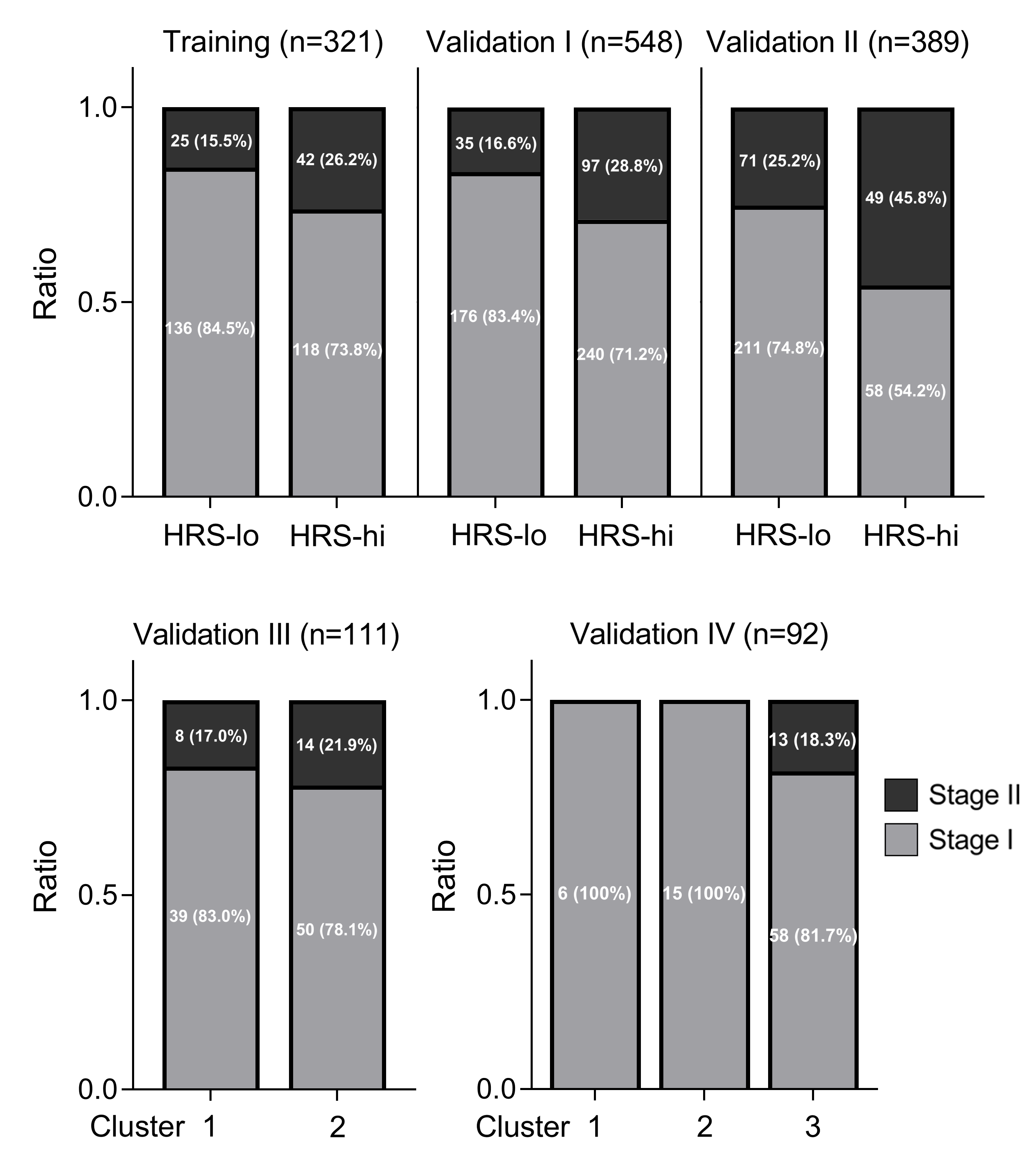

Supplement: Supplementary_figure_2 – Supplemental material for Development and validation of a hypoxia-related gene signature to predict overall survival in early-stage lung adenocarcinoma patients [file Supplementary_figure_2.tif]
